# Supplementary material for: An accessible, efficient and global approach for the large-scale sequencing of bacterial genomes
Source: Genome Biol. 2021 Dec 21;22:349. doi: 10.1186/s13059-021-02536-3 (PMC8690886; doi:10.1186/s13059-021-02536-3)
Supplement: Supplementary file 1 — Additional file 1: Includes supplementary figures S1, S2, and S3: Fig. S1. Average DNA concentration and molarity of libraries constructed using the LITE pipeline across individual 96-well plates. Fig. S2: Assessment of DNA integrity among libraries constructed using the LITE pipeline. Fig. S3: Integrity assessment of DNA extraction of 42 samples described in Table S2. [file 13059_2021_2536_MOESM1_ESM.pdf]

Fig. S1: Average DNA concentration and molarity of libraries constructed using the LITE pipeline across individual 96-well plates

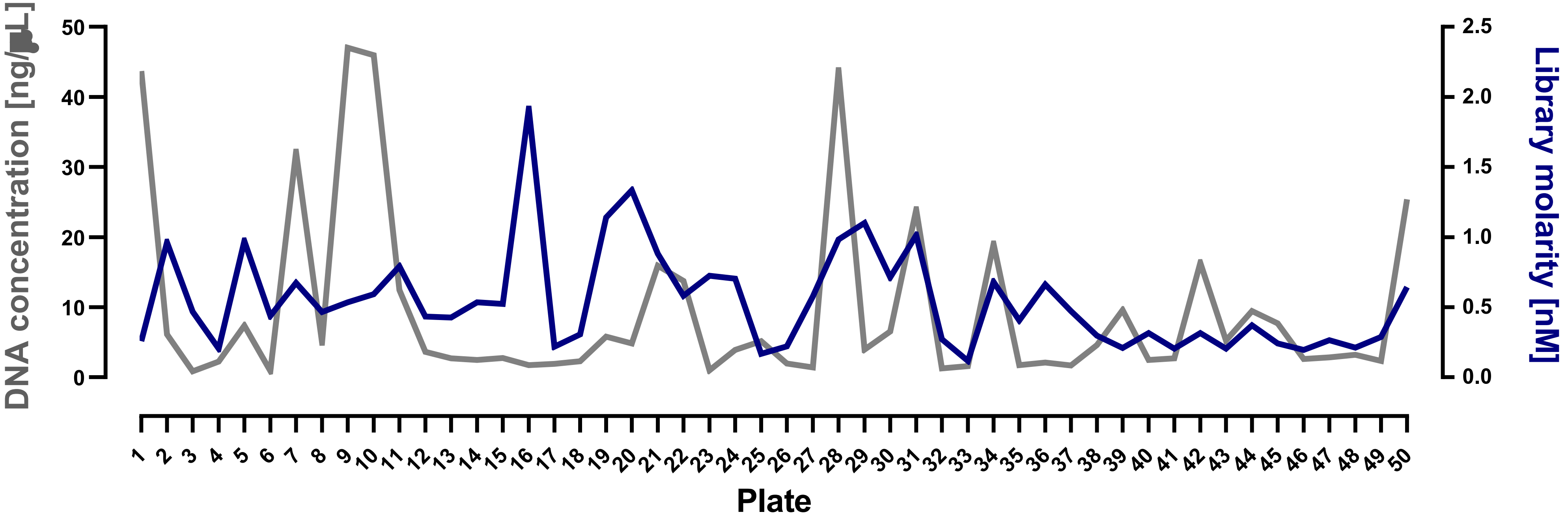

Fig. S2: Assessment of DNA integrity amongst libraries constructed using the LITE pipeline

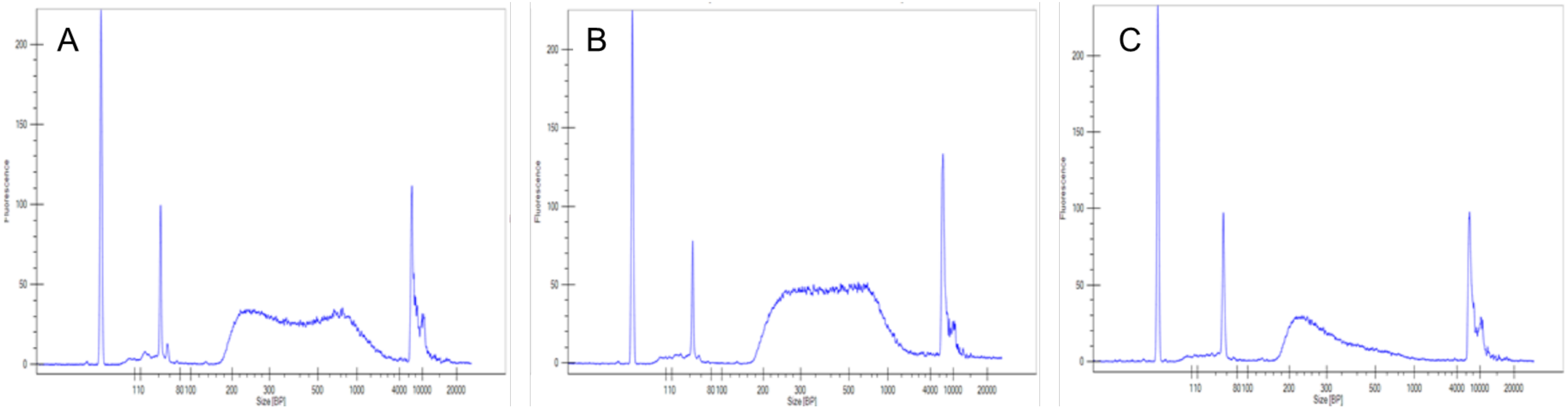

Fig. S3: Integrity assessment of DNA extraction of 42 isolates described in Supplementary Table 2

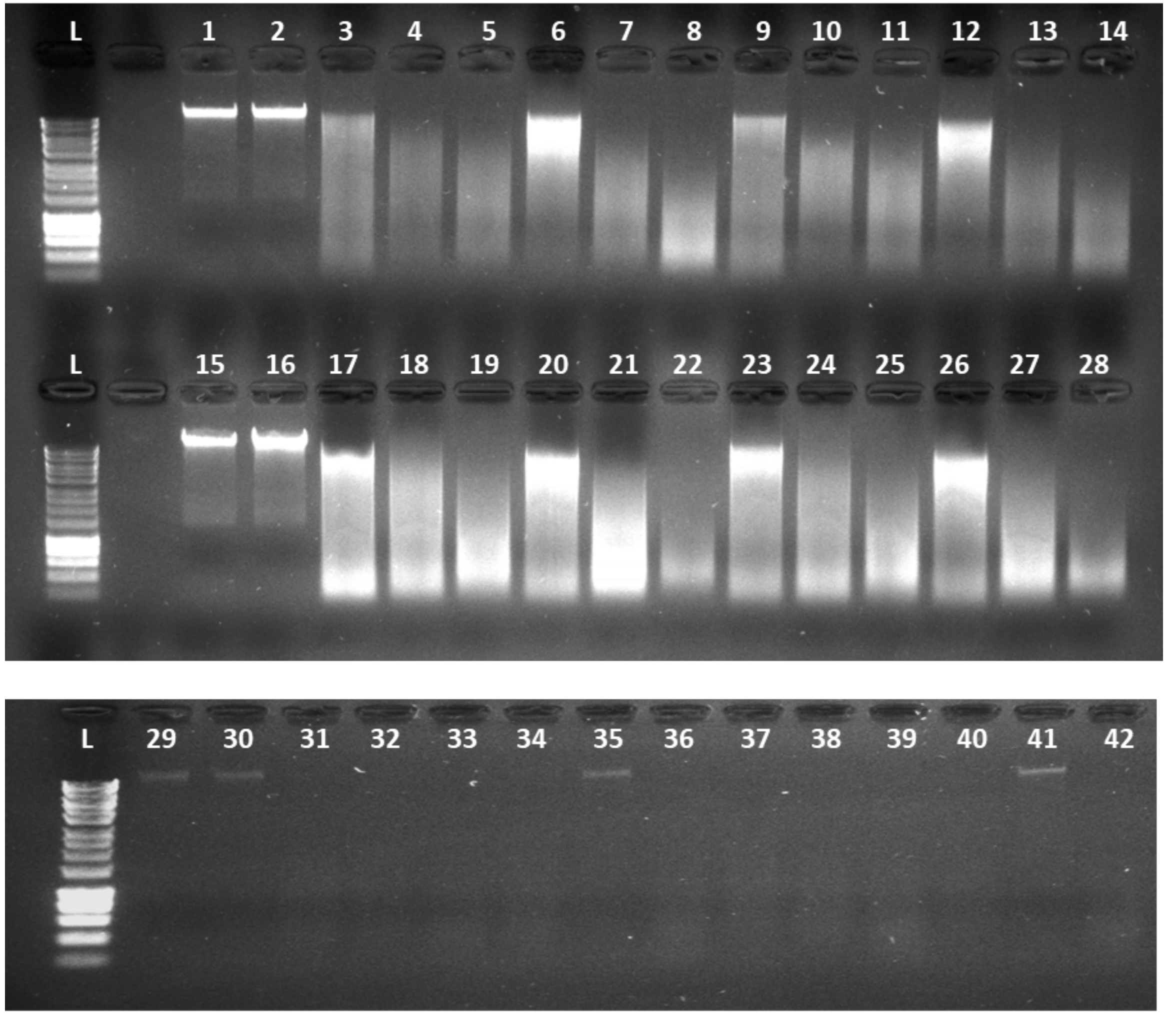

1% agarose/TAE (w/v) gel electrophoresis, run at 100 V for 30 min
L: HyperLadder 1 kb, Bioline

Lane	Sample	Temperature (°C)*	Time (min)*
1	*E. coli* K12	N/A	N/A
2	*E. coli* K12	N/A	N/A
3	*E. coli* K12	90	10
4	*E. coli* K12	95	10
5	*E. coli* K12	100	10
6	*E. coli* K12	90	20
7	*E. coli* K12	95	20
8	*E. coli* K12	100	20
9	*E. coli* K12	90	10
10	*E. coli* K12	95	10
11	*E. coli* K12	100	10
12	*E. coli* K12	90	20
13	*E. coli* K12	95	20
14	*E. coli* K12	100	20
15	*S. enterica* D23580	N/A	N/A
16	*S. enterica* D23580	N/A	N/A
17	*S. enterica* D23580	90	10
18	*S. enterica* D23580	95	10
19	*S. enterica* D23580	100	10
20	*S. enterica* D23580	90	20
21	*S. enterica* D23580	95	20

Lane	Sample	Temperature (°C)*	Time (min)*
22	*S. enterica* D23580	100	20
23	*S. enterica* D23580	90	10
24	*S. enterica* D23580	95	10
25	*S. enterica* D23580	100	10
26	*S. enterica* D23580	90	20
27	*S. enterica* D23580	95	20
28	*S. enterica* D23580	100	20
29	*S. aureus* Newman	N/A	N/A
30	*S. aureus* Newman	N/A	N/A
31	*S. aureus* Newman	90	10
32	*S. aureus* Newman	95	10
33	*S. aureus* Newman	100	10
34	*S. aureus* Newman	90	20
35	*S. aureus* Newman	95	20
36	*S. aureus* Newman	100	20
37	*S. aureus* Newman	90	10
38	*S. aureus* Newman	95	10
39	*S. aureus* Newman	100	10
40	*S. aureus* Newman	90	20
41	*S. aureus* Newman	95	20
42	*S. aureus* Newman	100	20

*N/A: not applicable
