# Supplementary material for: An accessible, efficient and global approach for the large-scale sequencing of bacterial genomes
Source: Genome Biol. 2021 Dec 21;22:349. doi: 10.1186/s13059-021-02536-3 (PMC8690886; doi:10.1186/s13059-021-02536-3)
Supplement: Supplementary file 3 — Additional file 3. Review history. [file 13059_2021_2536_MOESM3_ESM.docx]

Review History

**First round of review**

**Reviewer 1**

**Are you able to assess all statistics in the manuscript, including the appropriateness of statistical tests used?** Yes: The statistics are fine - this is more a paper about methods of high-throughput sequencing of bacterial genomes.

**Comments to author:**

Please consider the following questions:

- Are the methods appropriate to the aims of the study, are they well described, and are necessary controls included? YES.

- Are the conclusions adequately supported by the data shown? YES

- Are sufficient details provided to allow replication and comparison with related analyses that may have been performed? YES

- Is the method likely to be of broad utility? Is any software component easy to install and use? Please indicate briefly the novel features and/or advantages of the method, and/or please reference the relevant publications and which methods, if any, it should be compared with. YES.

The idea behind this paper is to develop methods for allowing high-throughput sequencing of bacterial genomes from third-world countries. There are a number of logistical problems, including transport without requiring refrigeration. The authors show that by heat inactivating the samples, they can be transported at room temperature. There are several other tricks that can be employed to allow collection of genomic DNA in 96 well microtiter plates. In the end, they could sequence 96 samples for about $1000, or close to $10 per sample. All the sequence reads have been deposited to the European Nucleotide Archive (ENA), and I could find them all, and had a look at a couple of them, and they appear to be publicly available, and easily found (with the project accession number).

- Is the paper of broad interest to others in the field, or of outstanding interest to a broad audience of biologists? YES - having a method for being able to sequence bacterial genomes for about $10 per sample is great! I think that LOTS of people would be interested in this.

**Reviewer 2**

**Are you able to assess all statistics in the manuscript, including the appropriateness of statistical tests used?** Yes: no statistics were undertaken due to the nature of the paper (methods)

**Comments to author:**

The manuscript by Perez-Sepulveda demonstrates to methodological discussion into how 10KSG consortium compiled genome sequences of "10,419" isolates primarily from Low income settings. The authors should be commended on tabulating a large database of genomes from low income countries. Basic frameworks are made available that includes access to generic bioinformatic scripts and sample metadata tables. The wider utility of this study is very much on the sequence database itself and less on the decision processes associated with initial method development aspects (eg extraction methods). The aspects pertaining to non-Salmonella aspects of data collection and metadata are weak/inadequately explained. As with all genome database publications, the research value of this study lies in the availability of metadata associated with the study, which in this study is lacking, despite being highlighted as a key criterion. Less convincing is the utility of the whole pipeline to the field wanting to undertake similar studies in other pathogens.

General comments:

1. The abstract states 10,419 isolates. This is contrary to what the actual outputs were: Sequence reads for 9,976 (96.0%); 7,236 were Salmonella (1,157 genomes from other species were included); 5,833 passed bioinformatic QC (6,117 after bioinformatic recovery). The Discussion (line 312) mentions "7,236 high-quality genomes" followed by the conclusion "generating 6,117 good-quality genomes". There needs to be a clear definition of what is meant by high-quality v good-quality. In this context, the "10,419" in the abstract is misleading as its not what is actually available.

2. The results for the non-Samonella genomes are not presented/discussed (or introduced for that matter). As these are included in this study as an important validation process, can the authors provide results into how their method development/interpretation applied to the other species.

3. Its unclear how this consortium differs to a parallel study that is publicly available at https://wellcomeopenresearch.org/articles/5-223/v1 that is another Salmonella 10k isolate study that encompasses overlapping geographical regions and also includes authors in the current consortia. While the welcome open research manuscript appears to be submitted after this manuscript by Perez-Sepulveda et al., the authors should provide a clear statement into how these studies overlap/differ.

4. As primarily a methods article, the isolate collation is vague in context (lines 327 to 336), and wider methodology should not be discussed "in brief" (line 338). No information is provided to the nature of the samples themselves. Were all isolates collated by microbiology labs within LIC? Was culturing practices standardised. How were environmental isolates identified? Line 338: Is this one colony from a clinical specimen. It appears as though some samples were treated differently (Lennox Broth or Buffered Peptone Water?) - which one was better. Why the difference? especially as the study is presenting a standardised methodology. How did this affect the different species?

5. As reviewers state, metadata is crucial and was a "crucial criterion for inclusion" (results paragraph 118 - 126). Why isnt key phenotypic data provided in Supp table 3. eg. source, sample type, Country, AMR etc. Such a rich data source needs to accompany this manuscript. The metadata table (supp table 3) only refers to Salmonella isolates. As such, no metadata is available for non-Salmonella isolates. As they are being reported in this study, they should be duly recorded and made publicly available. A number of isolates are also missing information. eg year and QC metrics. This information should be recorded in the metadata file for global utility. Submitting institutions should be recognised in this database as well.

6. Line 494 "Preliminary resistance and virulence gene profiling was done". No results are presented/discussed in this context. Why? Even as a way of confirming whether the phenotypic resistance profile matched the in silico profile (also helps address the plasmid query below)

7. As a methods article, the manuscript mentions that "we optimised a protocol for production of "thermolysates"", followed by "The optimisation involved testing under three different temperatures (90°C, 95°C or 100°C) and different incubation times (10 and 20 min)". The authors reference Supp table 2 which only contains "Quibit dsDNA HS (ng/mL)" values for only one of the 3 temperatures tested (95 degrees) are shown and only for some samples in once of the sample volumes and sample types (despite others being listed). Why? Values for other conditions tested should be recorded and a clear rationale for why 95 for 20 min was chosen. Based on this table, other conditions (eg 95deg for 10 minutes) looks to be more than adequate. Same query for all species tested/reported in this study.

8. DNA extraction line 375" DNA extraction using an optimised protocol based on the MagAttract HMW DNA isolation kit". What is the optimised protocol? Also, regarding line 380. Suspension was spun to 4k rpm and supernatant discarded. Considering that all samples were treated at 95 degrees for 20 min leading to lysis of cell, wouldnt the vast majority of DNA be in the supernatant at this stage and subsequently lost following centrifugation? This would provide an explanation why DNA retrieval and subsequence sequence failure was relatively high. Furthermore, how does the DNA extraction process and size exclusion parameters affect plasmids? These are key elements for AMR surveillance (a key goal).

9. "To allow transport without refrigeration, we tested the stability of the resulting thermolysates at room temperature for more than seven days by controlling the quality of extracted DNA (Supplementary Table 2)". There is no information reported in this table how results are affected by refrigeration over time. Can the authors explain how they can to this conclusion using the data provided?

10. Read coverage for this study appears low with 10% had <10x coverage. As stated, the target was "median 30x genome coverage". How many isolates passes this threshold? What was the range of coverage (provide plots). Does the low coverage in general actually indicate that the LITE method was actually sub-optimal? The study mentions that some (unknown) samples were provided in pre-extracted form. Was there a difference in outcome for samples provided in pre-extracted DNA form versus thermolysates?

11. Related to the previous comment, there is no detailed results/discussion provided relating to whether there was geographical variation in thermolysate quality between different settings or what the learnings were regarding the ~20% of the initiate 10,419 that failed. More informed discussion around what sample processing learnings would be beneficial, especially in the context of what didnt work.

12. While a github link is provided relating to scripts used, the scripts are not 'packaged' and there is not installation guide. As such, the bioinformatic workflow is not of sufficient detail for broad utility (unless a user has extensive bioinformatic skills). If the authors are proposing that this informatic pipelines be utilised by the wider community then packaging and/or step-by-step installation and code needs to be made available. The genome assemblies should be made publicly available (becoming more widely accepted) to enhance utility of database outside of bioinformatic savvy laboratories.

Minor comments:

13. No ethics statement provided. I would have thought that each country would require consent/approval in some context for this kind of study.

14. pg 5 line 124. Regarding phenotypic resistance. were all isolates been processed to the same guidelines? Details are missing and should be provided.

15. pg 7/ To validate this approach for bacteria other than Salmonella, ~25% (2,573, 24.7%) of the samples were isolates from a variety of genera, including Gram-negatives such as Shigella and Klebsiella, and Gram-positives such as Staphylococcus.

16. Table 1. I presume these prices are for 95 isolates. Add a cost/genome summary

17. line 249. what versions of tools? why unicycler which was designed for hybrid read assembly (long-reads and short-reads)? spades/shovill are more widely adapted.

18. pg 8 line 181. what was the concentration range and averages for collaborators supplying thermolysates v DNA extracts (suppl figure 1). How did both approaches relate to sequencing failure rate. What's the take-home message?

19. What was the base calling quality like? How does the proposed method deal with heteropolymeric SNPs? Other QC metrix for the dataset should be plotted and supplied.

20. Figure 4 bubble plot. break into blood/stool. The predominant portion of America serotypes are 'other'. Why? Rational for inclusion was SEN or STM, unclear why there were so many others.

21. publicly spelling : pg 4 line 79

22. pg 8, line 185 "; Fig 2)"

23. line 259 "enteritca"

24. line 276. in silico AMR profile derived?

25. line 345. "Eschericchia" spelling

26. line 391 "wae"

27. line 479 "a the"

28. line 489 "a a"

29. line 640 ". Hinton."

30. library construction (from line 404) what version of Nextera chemistry/kit? Add version throughout

31. line 482/483. What is meant by "in our case". State what was used.

32. line 490. Is the "custom protein database" being made publicly available (github?)

33. PRJEB35182 on ena shows 6086 samples. where's the rest? All samples need to be made publicly available.

**Authors Response**

**Point-by-point responses to the reviewers’ comments:**

**Reviewer #1:**

- Are the methods appropriate to the aims of the study, are they well described, and are necessary controls included?

YES.

- Are the conclusions adequately supported by the data shown?

YES

- Are sufficient details provided to allow replication and comparison with related analyses that may have been performed?

YES

- Is the method likely to be of broad utility? Is any software component easy to install and use? Please indicate briefly the novel features and/or advantages of the method, and/or please reference the relevant publications and which methods, if any, it should be compared with.

YES. The idea behind this paper is to develop methods for allowing high-throughput sequencing of bacterial genomes from third-world countries. There are a number of logistical problems, including transport without requiring refrigeration. The authors show that by heat inactivating the samples, they can be transported at room temperature. There are several other tricks that can be employed to allow collection of genomic DNA in 96 well microtiter plates. In the end, they could sequence 96 samples for about $1000, or close to $10 per sample. All the sequence reads have been deposited to the European Nucleotide Archive (ENA), and I could find them all, and had a look at a couple of them, and they appear to be publicly available, and easily found (with the project accession number).

- Is the paper of broad interest to others in the field, or of outstanding interest to a broad audience of biologists?

YES - having a method for being able to sequence bacterial genomes for about $10 per sample is great! I think that LOTS of people would be interested in this.

We agree that the development of the ability to generate bacteria genomes for just $10 was a key motivation behind the study. We anticipate that this factor is likely to generate a lot of interest in our approach in the wider public health and microbiological communities, particularly in low and middle-income countries.

Reviewer #2:

The manuscript by Perez-Sepulveda demonstrates to methodological discussion into how 10KSG consortium compiled genome sequences of "10,419" isolates primarily from Low income settings. The authors should be commended on tabulating a large database of genomes from low income countries. Basic frameworks are made available that includes access to generic bioinformatic scripts and sample metadata tables. **The wider utility of this study is very much on the sequence database itself and less on the decision processes associated with initial method development aspects (eg extraction methods).**

We respectfully disagree with the reviewer. A key reason for the success of the 10KSG project was the novel strategy we used for shipping bacterial samples from low-income countries. The approach involved the generation of thermolysates (heat-killed bacterial cultures) which were subsequently shipped internationally at ambient temperatures. It was essential that dry ice shipments were avoided. Dry ice is both expensive and rare in resource-limited settings in Africa.

The aspects pertaining to non-Salmonella aspects of data collection and metadata are weak/inadequately explained.

We agree that the submitted version of the manuscript was highly-focused on *Salmonella*. As this was both a method and a resource paper, certain non-*Salmonella* aspects of data collection and metadata had not been highlighted.

We have improved the revised version of the manuscript by expanding the data provided in Supplementary Table 3 to **~98% of sequenced isolates** (9,746 out of 9,976 sequenced isolates). These include all of the sequence data available for bacteria of seven genera, *Acinetobacter*, *Enterobacter, Klebsiella, Pseudomonas, Shigella*, and *Staphylococcus* and *Salmonella* (including the serovars Typhimurium, Enteritidis, Agona, Dublin, Heidelberg, and others).

As with all genome database publications, the research value of this study lies in the availability of metadata associated with the study, which in this study is lacking, despite being highlighted as a key criterion.

We completely understand the importance of the metadata. However, for this type of proof-of-concept study, we are constrained by contractual agreements with our collaborators from Low-Middle-Income-Countries which specify that the metadata will only be released when the key scientific findings from the study are published.

As an example of our approach, the analysis of 680 African *S.*Typhimurium genomes was published in Nature Microbiology on 21^st^ December 2020 (<https://www.nature.com/articles/s41564-020-00836-1>, DOI: 10.1038/s41564-020-00836-1), and ALL the associated metadata have already been uploaded to the ENA. The associated accession numbers are detailed in Table S2.

Less convincing is the utility of the whole pipeline to the field wanting to undertake similar studies in other pathogens.

We respectfully disagree with the reviewer. As we explained in our covering letter, access to the game-changing genome sequencing technology for scientists in low- and middle-income countries has remained restricted. It was the need to “democratise” the field of bacterial genomic analysis that prompted us to develop our new strategy to allow collaborators based in many economically-challenged countries to contribute to the sequencing of thousands of bacterial isolates. We predict that our pipeline will be useful to the worldwide research community.

General comments:

1. The abstract states 10,419 isolates. This is contrary to what the actual outputs were: Sequence reads for 9,976 (96.0%); 7,236 were Salmonella (1,157 genomes from other species were included); 5,833 passed bioinformatic QC (6,117 after bioinformatic recovery). The Discussion (line 312) mentions "7,236 high-quality genomes" followed by the conclusion "generating 6,117 good-quality genomes". There needs to be a clear definition of what is meant by high-quality v good-quality. In this context, the "10,419" in the abstract is misleading as its not what is actually available.

We agree this is an important point, and have added a more detailed explanation the “high vs good quality” numbers in the revised version of the manuscript. We respectfully point out that it was not misleading to state that we began by processing 10,419 isolates at the start of the project, as the manuscript explained how the 10,419 bacterial genomes were whittled down to 6,117 high-quality *Salmonella* genomes (Figure 3). We have added these important clarifications to the text, and updated Figure 3.

2. The results for the non-Salmonella genomes are not presented/discussed (or introduced for that matter). As these are included in this study as an important validation process, can the authors provide results into how their method development/interpretation applied to the other species.

We are grateful for the reviewer’s suggestion, and agree that it will be valuable to include validation for the non-Salmonella genomes. We have now improved the revised manuscript by have expanding the data provided in Supplementary Table 3 to cover ~98% of the sequenced isolates (9,746 out of 9,976 sequenced isolates).

3. Its unclear how this consortium differs to a parallel study that is publicly available at <https://wellcomeopenresearch.org/articles/5-223/v1> that is another Salmonella 10k isolate study that encompasses overlapping geographical regions and also includes authors in the current consortia. While the welcome open research manuscript appears to be submitted after this manuscript by Perez-Sepulveda et al., the authors should provide a clear statement into how these studies overlap/differ.

We are happy to explain how the parallel *Salmonella* 10k isolate study differs from our project – this work from Mark Achtman’s group, published in Wellcome Open Research after our work was published in BioRxiv, complements our work extremely well. It focuses on a diverse range of *Salmonella* isolates from the environment, from animals and from human stool. These isolates originated from a limited geographical region with a major focus on Ireland.

In contrast, our study had a global focus, with the majority of the *Salmonella* isolates obtained from the human bloodstream of patients in Africa and Latin America.

We have added a paragraph to the manuscript to add this important context:
“Indeed, a recent study by Achtman *et al*. (2020) [17] sequenced 9,591 *Salmonella* genomes isolated mainly from water and animal sources in the USA, Europe and Taiwan. This collaboration between the University of Warwick (UK) and the University College Cork (Ireland) focused on the analysis of three serovars, and added an important level of diversity to the publicly available genomes for the *Salmonella* community, which were mostly obtained from food of human isolates.”

4. As primarily a methods article, the isolate collation is vague in context (lines 327 to 336), and wider methodology should not be discussed "in brief" (line 338). No information is provided to the nature of the samples themselves. Were all isolates collated by microbiology labs within LIC? Was culturing practices standardised. How were environmental isolates identified? Line 338: Is this one colony from a clinical specimen. It appears as though some samples were treated differently (Lennox Broth or Buffered Peptone Water?) - which one was better. Why the difference? especially as the study is presenting a standardised methodology. How did this affect the different species?

We are grateful to the reviewer for pointing this out, and have clarified these points in the revised version of the manuscript.

5. As reviewers state, metadata is crucial and was a "crucial criterion for inclusion" (results paragraph 118 - 126). Why isnt key phenotypic data provided in Supp table 3. eg. source, sample type, Country, AMR etc. Such a rich data source needs to accompany this manuscript. The metadata table (supp table 3) only refers to Salmonella isolates. As such, no metadata is available for non-Salmonella isolates. As they are being reported in this study, they should be duly recorded and made publicly available. A number of isolates are also missing information. eg year and QC metrics. This information should be recorded in the metadata file for global utility. Submitting institutions should be recognised in this database as well.

As mentioned above, we completely understand the importance of the metadata. However, we are constrained by the contractual agreements with our collaborators from Low-Middle-Income-Countries which specifies that the metadata will only be released when the key scientific findings from specific parts of the study are published.

We have provided a new Table 1 that summarises the key characteristics of the isolates from the 10KGSP, namely sample origin, source, body compartment, and antimicrobial resistance.

6. Line 494 "Preliminary resistance and virulence gene profiling was done". No results are presented/discussed in this context. Why? Even as a way of confirming whether the phenotypic resistance profile matched the in silico profile (also helps address the plasmid query below)

We must emphasise that this is primarily a Methods pape. The details concerning the profiles of resistance and virulence genes will be published with individual research studies that are currently in progress.

Since our original submission to Genome Biology, we have published or submitted four research papers that describe the relationship between phenotypic resistance profiles *and in silico*-derived profiles for *Salmonella* isolates that were genome-sequenced as part of the 10KGSP:

- Our analysis of 680 African *S.* Typhimurium genomes (published in Nature Microbiology (Pulford *et al.,* 2021 <https://www.nature.com/articles/s41564-020-00836-1>), includes the detailed phenotypic comparison of the antibiotic susceptibility phenotypes of 480 isolates with the genome-based AMR predictions.
- Kasumba *et al.,* 2021 Clin. Infect. Dis. <https://doi.org/10.1093/cid/ciab051>
- Wilson et al., 2020 PloS Neg. Trop Dis <https://doi.org/10.1371/journal.pntd.0008796>
- Darboe et al., 2021 BioRxiv [**https://www.biorxiv.org/content/10.1101/2020.10.30.316588v1**](https://www.biorxiv.org/content/10.1101/2020.10.30.316588v1)

These four papers are now referenced in the revised manuscript.

7. As a methods article, the manuscript mentions that "we optimised a protocol for production of "thermolysates"", followed by "The optimisation involved testing under three different temperatures (90°C, 95°C or 100°C) and different incubation times (10 and 20 min)". The authors reference Supp table 2 which only contains "Qubit dsDNA HS (ng/mL)" values for only one of the 3 temperatures tested (95 degrees) are shown and only for some samples in one of the sample volumes and sample types (despite others being listed). Why? Values for other conditions tested should be recorded and a clear rationale for why 95 for 20 min was chosen. Based on this table, other conditions (eg 95deg for 10 minutes) looks to be more than adequate. Same query for all species tested/reported in this study.

We are pleased to clarified this important point in the revised manuscript.

We have performed electrophoretic validation of many more DNA samples, and have added these data to the supplementary information of the revised version.

The reason that a 20-minute period of treatment at 95° was chosen was to ensure that dangerous pathogens were effectively killed by the protocol, as recommended by our Biological Safety Office. The DNA extraction and genome-sequencing of the different pathogens tested were successful using those parameters, as detailed in the revised manuscript.

Accordingly, we have provided more data on DNA extraction assessment in Supplementary Table 2 and a new Supplementary Figure 3.

8. DNA extraction line 375" DNA extraction using an optimised protocol based on the MagAttract HMW DNA isolation kit". What is the optimised protocol? Also, regarding line 380. Suspension was spun to 4k rpm and supernatant discarded. Considering that all samples were treated at 95 degrees for 20 min leading to lysis of cell, wouldnt the vast majority of DNA be in the supernatant at this stage and subsequently lost following centrifugation? This would provide an explanation why DNA retrieval and subsequence sequence failure was relatively high. Furthermore, how does the DNA extraction process and size exclusion parameters affect plasmids? These are key elements for AMR surveillance (a key goal).

We are grateful to the reviewer for pointing out this omission. We have now added more text to this section to clarify the amendments to the MagAttract method. We now highlight the fact that pellets were observed following centrifugation, ensuring that sufficient intact material entered the DNA extraction pipeline.

Here is the revised text:

“DNA was extracted from bacterial thermolysates on a Biomek FXP instrument using a reduced volume protocol of the MagAttract HMW DNA isolation kit (Qiagen). Incomplete barcoded 96-tube plates received were re-organised and FluidX barcodes re-read using the FluidX barcode reader and software prior to DNA extraction, to determine plate layouts. The tubes were de-capped using a manual eight-tube decapper and the cellular material was re-suspended using a multichannel pipette. Up to 100 µL of the suspension were transferred to a clean 96-well plate. The plate was spun at 4,000 rpm in an Eppendorf 5810R centrifuge and visible pellets were observed in a majority of cases indicating the presence of cellular material. Plates were then upturned and the supernatant carefully discarded.”

9. "To allow transport without refrigeration, we tested the stability of the resulting thermolysates at room temperature for more than seven days by controlling the quality of extracted DNA (Supplementary Table 2)". There is no information reported in this table how results are affected by refrigeration over time. Can the authors explain how they can to this conclusion using the data provided?

We are grateful for the opportunity to improve this aspect of the paper, and have provided more data related to DNA extraction in the revised version of the manuscript. The data are now included in Supplementary Table 2 and a new Supplementary Figure 3.

10. Read coverage for this study appears low with 10% had <10x coverage. As stated, the target was "median 30x genome coverage". How many isolates passes this threshold? What was the range of coverage (provide plots). Does the low coverage in general actually indicate that the LITE method was actually sub-optimal? The study mentions that some (unknown) samples were provided in pre-extracted form. Was there a difference in outcome for samples provided in pre-extracted DNA form versus thermolysates?

We are grateful that the reviewer highlighted this important point, which we have addressed in the revised manuscript. Overall, 57.6% of isolates passed the "median 30x genome coverage" threshold, details are now included in Supplementary Table 3.

The LITE method represents an extremely cost-efficient way to sequence 10,000 bacterial genomes, and achieved the <USD10 cost-per-genome by using relatively low levels of genome coverage. Our manuscript shows that this level of genome coverage was sufficient for most analytical approaches.

We have elaborated on this important issue in the revised manuscript, and have added a section that details the “advantages and limitations of the study” to the Discussion.

Unfortunately, we are not able to determine whether there was a difference in outcome for samples provided as pre-extracted DNA.

11. Related to the previous comment, there is no detailed results/discussion provided relating to whether there was geographical variation in thermolysate quality between different settings or what the learnings were regarding the ~20% of the initiate 10,419 that failed. More informed discussion around what sample processing learnings would be beneficial, especially in the context of what didnt work.

Clearly, we did not explain this carefully enough. In fact, 20% of samples did not fail sequencing. Only **4.3%** of samples failed sequencing. Our analysis did not identify any geographical variation from thermolysates. We have elaborated on this point in the “advantages and limitations of this study” section as follows:

Line 312: “An advantage of the LITE pipeline is the low DNA requirement. For *Salmonella*, 1 ng equates to >150,000 copies of the genome, making this a robust pipeline that captures all the genetic material. Also, production of thermolysates helped increase throughput as it facilitated automated DNA extraction in a central location, allowing many collaborators to provide samples without needing to extract DNA. This optimised DNA extraction and sequencing pipeline, in conjunction with the generation of thermolysates, provides a robust approach for global collaboration on the genome-based mass surveillance of pathogens.”

12. While a github link is provided relating to scripts used, the scripts are not 'packaged' and there is not installation guide. As such, the bioinformatic workflow is not of sufficient detail for broad utility (unless a user has extensive bioinformatic skills). If the authors are proposing that this informatic pipelines be utilised by the wider community then packaging and/or step-by-step installation and code needs to be made available. The genome assemblies should be made publicly available (becoming more widely accepted) to enhance utility of database outside of bioinformatic savvy laboratories.

We are grateful for the reviewer's suggestion, and have updated the github to include version numbers of all software, and the Directory Structure that will allow installation anywhere in the world. We agree that, the installation will require some bioinformatic expertise. The development of a broader bioinformatic pipeline that could be used by scientists with less computational experience would be a significant project in itself and is, unfortunately, beyond the scope of our study.

The genomic data are provided in the European Nucleotide Archive (accession no PRJEB35182), and detailed in Supplementary Table 5. Genome assemblies will be made available when the individual research studies that are currently in progress are published.

Minor comments:

13.	No ethics statement provided. I would have thought that each country would require consent/approval in some context for this kind of study.

We are grateful for the opportunity to address this important issue. No ethics statements were required because the project did not involve live bacterial cultures. We have added clarification to the revised manuscript, and have stated that the “Production of thermolysates permitted simplification of material transfer agreements (MTAs) as there was no distribution of live cultures.”

Details of the Material Transfer Agreements that underpin the project have been added to the revised version of the manuscript.

14.	pg 5 line 124. Regarding phenotypic resistance. were all isolates been processed to the same guidelines? Details are missing and should be provided.

We are grateful for the opportunity to clarify this important point about metadata collection and standardisation, and have added the following text to the revised version of the manuscript:

“We added an extra column to include relevant information that could not be assigned to any other category, such as type of study and references. The metadata collected were stored per collaborator, and then combined into a metadata master form for curation. Curation was done manually, standardising each category by column while maintaining version control. The final metadata master form (Supplementary Table 3) was cross-referenced with the list of barcodes that had been sent to individual collaborators to identify any inconsistencies.”

15.	pg 7/ To validate this approach for bacteria other than Salmonella, ~25% (2,573, 24.7%) of the samples were isolates from a variety of genera, including Gram-negatives such as Shigella and Klebsiella, and Gram-positives such as Staphylococcus.

We have clarified this point in the revised version of the manuscript.

16.	Table 1. I presume these prices are for 95 isolates. Add a cost/genome summary

The cost/genome summary is now included in the revised manuscript, at Line 174:
“The total consumable cost for extraction of DNA and genome sequence generation was less than USD$10 per sample (excluding staff time).”

17.	line 249. What versions of tools? Why unicycler which was designed for hybrid read assembly (long-reads and short-reads)? Spades/shovill are more widely adapted.

The details for all versions have been added to the manuscript, and can be found in <https://github.com/apredeus/10k_genomes>.

We agree that SPAdes is an established and widely-used tool for bacterial genome assembly. Unicycler builds on the SPAdes assembly by optimising SPAdes parameters, and subsequently doing “assembly polishing” by mapping reads back to the assembled genomes. Unicycler is a “next generation” assembly tool that is being increasingly adopted across the bacterial genomic community.

18.	pg 8 line 181. what was the concentration range and averages for collaborators supplying thermolysates v DNA extracts (suppl figure 1). How did both approaches relate to sequencing failure rate. What's the take-home message?

Our experience with the LITE protocol has taught us that DNA integrity is the biggest factor in success/ failure of successful DNA library construction, and highly degraded DNA is more likely to result in a failed library.

Unfortunately, we do not have the information requested by the reviewer as the accurate determination of the molecule size of each sample would more than double the cost of project, which was intended to be a low-cost pipeline.

The data we do have available show a wide overlap between DNA yields and outcomes, both within the thermal lysates and the supplied DNA. For example, of 282 thermo lysates which yielded DNA between 0.1 and 0.2ng/ µl only 27 failed to generate sequence data. At the same time, a couple of samples that yielded >100ng/ µl failed to generate any sequence data.

Accordingly, we do not yet have a clear take-home message regarding the link between DNA concentration and sequencing failure.

We have now assessed this important issue in the “advantages and limitations of this study” section in the Discussion.

19.	What was the base calling quality like? How does the proposed method deal with heteropolymeric SNPs? Other QC metrix for the dataset should be plotted and supplied.

This issue has been addressed in the revised version of the manuscript.

20.	Figure 4 bubble plot. break into blood/stool. The predominant portion of America serotypes are 'other'. Why? Rational for inclusion was SEN or STM, unclear why there were so many others.

The reviewer is correct that our “rationale for inclusion” was that the *Salmonella* isolates isolates were either Enteritidis or Typhimurium, and that majority of the American samples are *Salmonella* Enteritidis. However, due to the technical limitations experienced by laboratories in low-to-middle-income countries, it was inevitable that a small number of the Salmonella isolates would represent different serovars. Because the American *Salmonella* isolates included 70 different serovars, the individual serovars were not highlighted in the text, and have been grouped as “other”.

21.	publicly spelling : pg 4 line 79

22.	pg 8, line 185 "; Fig 2)"

23.	line 259 "enteritca"

24.	line 276. in silico AMR profile derived?

25.	line 345. "Eschericchia" spelling

26.	line 391 "wae"

27.	line 479 "a the"

28.	line 489 "a a"

29.	line 640 ". Hinton."

30.	library construction (from line 404) what version of Nextera chemistry/kit? Add version throughout

31.	line 482/483. What is meant by "in our case". State what was used.

32.	line 490. Is the "custom protein database" being made publicly available (github?)

33.	PRJEB35182 on ena shows 6086 samples. where's the rest? All samples need to be made publicly available.

We are grateful for the opportunity to fix these issues. Points 21 to 33 have been addressed in the revised version of the manuscript.

**Second round of review**

**Reviewer 2**

The reviewed manuscript does clarify a number of points raised, demonstrating that the methodology employed by the '10KSG consortium' resulted in genomes of ~10k bugs from LMIC for ~$10US/sample. As pointed out, this does not include staffing for all nodes which rapidly inflates the actual cost. The bioinformatics pipeline is also not stand-alone which is not surprising, yet impacts broader implementation. The authors did partially address the issue of metadata: Table S3 is an important improvement yet it would be great to see assemblies submitted to a public repository considering that the authors are reporting assembly statistics as validation of their approach.

In the revised version: Its not clear where 'Q4' was addressed: "Was culturing practices standardised? How were environmental isolates identified? Line 338: Is this one colony from a clinical specimen. It appears as though some samples were treated differently (Lennox Broth or Buffered Peptone Water?) - which one was better. Why the difference? especially as the study is presenting a standardised methodology. How did this affect the different species?"

Can the authors state where these queries were addressed? Q6: "The details concerning the profiles of resistance and virulence genes will be published with individual research studies that are currently in progress." If such results are not going to be presented with this publication then the authors should not state that "preliminary resistance and virulence gene profiling was done" (eg line 528 and inferred in other sections) within this manuscript. Yes - the overall numbers of MDR have been added to Table 1, but nothing is reported on virulence. If assembly stats are to be documented (table S3), can the consortia submit these assemblies to public repositories (which is becoming a standard process)? Adding the virulence and resistance data to this table would also support their statements of preliminary resistance and virulence gene profiling.

**Authors Response**

**Point-by-point responses to the reviewers’ comments:**

**Responses to Reviewers**

It’s not clear where 'Q4' was addressed:

"Was culturing practices standardised? How were environmental isolates identified? Line 338: Is this one colony from a clinical specimen. It appears as though some samples were treated differently (Lennox Broth or Buffered Peptone Water?) - which one was better. Why the difference? especially as the study is presenting a standardised methodology. How did this affect the different species?"

We are grateful to the reviewer for requesting this important clarification to answer Q4.

During this project, culturing practices were standardised to use the same volume, time, and temperature of incubation. The inoculum was defined as either a “scoop” or a bead collected with a sterile inoculation loop from a frozen stock prepared from a single bacterial colony.

Environmental isolates of *Salmonella* bacteria were identified and processed in the same way as clinical and animal isolates, at the reference laboratories and research institutions providing the isolates.

However, some of the research institutions involved in this worldwide collaborative project used Lennox Broth whereas others relied on Buffered Peptone Water media. Both are rich, non-selective media that permitted the growth of the biomass required to extract genomic DNA for sequencing. The two media are routinely used in diagnostic microbiology laboratories to cultivate *Salmonella*, *Escherichia* and *Staphylococcus* bacteria (Baylis, McPhee & Betts. 2000. *J. Appl. Microbiol.*89(3):501-10; Perry-O’Keefe *et al.* 2001. *J. Microbiol. Methods.*47(3):281-92). Similar levels of genomic DNA were isolated from bacterial cultures grown in both media (Supplementary Figure S1).

We have clarified the key points in the following paragraph:

“Method optimisation focused on standardising a safe protocol for sample transport and processing. The optimised method comprised bacterial isolates grown at 37°C overnight directly in FluidX tubes (FluidX tri-coded jacket 0.7 mL, 68-0702-11, Brooks Life Sciences) that contained 100 µL rich media, and were inoculated from a frozen stock prepared from one bacterial colony (one “scoop” or bead (Microbank^TM^, Pro Lab Diagnostics Inc.)). The rich media used in the reference laboratories involved in this large collaborative study were either Lennox Broth or Buffered Peptone Water, depending on the protocols used in different laboratory settings. After overnight growth, the bacterial samples were inactivated by incubation at > 95°C for 20 min, followed by storage at 4°C until collection. Sample transportation was carried out at ambient temperature.

To ensure that the heat-inactivation step had the expected effect on organism viability, a controlled experiment was performed. Three bacterial strains were tested, namely ~~method using~~ *Salmonella enterica* serovar Typhimurium D23580, *Escherichia coli* K12, and *Staphylococcus aureus* Newman. Either a “scoop” with a 10 µL plastic loop taken from a bacterial glycerol (50% v/v) stock or 2 beads of bacteria stored at -80°C in a Microbank tube™ cryotubes (Pro-Lab Diagnostics) were used as inocula. The samples were grown at 37°C and 220 rpm overnight in either 100 or 200 µL LB (1% tryptone, 0.5% yeast extract, 0.5% NaCl; pH 7.0) in FluidX tubes. The effect of three temperatures and two times upon microbial viability was determined as follows:

100 µL of each sample was heated to either 90°C, 95°C or 100°C for 10 min or 20 min, and then plated on nutrient agar (1.5% agar LB (w/v)) for CFU determination (Supplementary Table 2).”

“Q6: "The details concerning the profiles of resistance and virulence genes will be published with individual research studies that are currently in progress." If such results are not going to be presented with this publication then the authors should not state that "preliminary resistance and virulence gene profiling was done (eg line 528 and inferred in other sections) within this manuscript.” Yes - the overall numbers of MDRhave been added to Table 1, but nothing is reported on virulence.”

We have split Table 1 to clarify numbers for the MDR and added to following lines to give more information to the reader

"Our analysis of a sample of 680 S. Typhimurium isolates showed that

genome-based analysis accurately predicted the AMR phenotypes of 89.8 % isolates,

with 87-98% sensitivity (86.7% chloramphenicol, 98.0% ampicillin, 97.4%

cotrimoxazole) and 77-96% specificity (83.6% chloramphenicol, 95.6% ampicillin,

77.1% cotrimoxazole) [34]."

we believe this information is important to give the reader a full description of the dataset generated.
